# McKillip’s Loss

**Published:** 1896-02

**Authors:** 


### McKILLIP'S LOSS.

THE severance of Prof. Schwarzkopf's relation with the McKillip Veterinary College will be a surprise and a great disappointment to many of our readers. His loss to that school will be felt in many ways. First because he associated himself therewith at a peculiar time in the history of veterinary education in America, and brought to its support all the enthusiasm and earnestness that has ever characterized him as an advocate of higher veterinary education in America. Again, of all those associated with this school in the capacity of teachers, there was none so well known among the leaders of advanced education in America as Prof. Schwarzkopf, and this strength, this added force, this influence he carried with him in all its power, and placed McKillip on the high plane she occupies to-day. We shall not attempt to pass upon the many causes that are credited with leading to this rupture, but we earnestly deplore the result that has culminated in this change. We shall not for a moment lose our interest or warm feeling for this school, but in every way support it, so long as we feel that this has been done for the promulgation of a better effort to win for McKillip a higher place among the colleges. All such dissensions in the early history of any school are disastrous, demoralizing, and far-reaching in injurious effects, and time only will tell whether the lesser or greater evil will prevail from this much-to-be-regretted change.

